# Corneoscleral graft in Mooren's ulcer: a case report

**DOI:** 10.1186/1757-1626-2-180

**Published:** 2009-11-02

**Authors:** Mauro Cellini, Michela Fresina, Ernesto Strobbe, Corrado Gizzi, Emilio C Campos

**Affiliations:** 1 Department of Surgery Science and Anesthesiology University of Bologna, Ophthalmology Service, Via Palagi 9 - 40138 Bologna, Italy

## Abstract

**Introduction:**

Mooren's ulcer is a rare disorder of unknown etiology that is refractory to treatment. It can affect not just the cornea but also the scleral tissue and can involve both eyes.

**Case presentation:**

We report a case of a 74-year-old man with a history of bilateral and malignant Mooren's ulcer. The patient had undergone an exenteratio bulbi of the left eye because of the perforation of a Mooren's corneal ulcer. The perforated Mooren's corneal ulcer also presented in the right eye and involved the adjacent scleral tissue. It was decided to perform a corneal-scleral graft to preserve the anatomical integrity of the eye.

**Conclusion:**

This report highlights how a corneal-scleral graft followed by systemic and local immunosuppressive treatment should be considered in monocular patients with malignant Mooren's ulcer where there is serious damage to the corneal and scleral tissue.

## Introduction

Mooren's ulcer is a rare disorder involving the chronic and painful ulceration of the cornea. The lesion with overhanging edges generally starts on the periphery and tends to spread progressively to the entire circumference or towards the centre of the cornea. As well as the cornea, the sclera can also be involved with an incidence of 13.5% of eye perforation and loss of vision [1]. When not only the cornea but also the sclera is involved in eye perforation it can be necessary to perform a large corneoscleral graft to save the eye. We describe a case of anterior segment reconstruction using corneoscleral grafts of 14 mm in diameter in a patient with Mooren's ulcer and eye perforation.

## Case presentation

**Figure 1:**
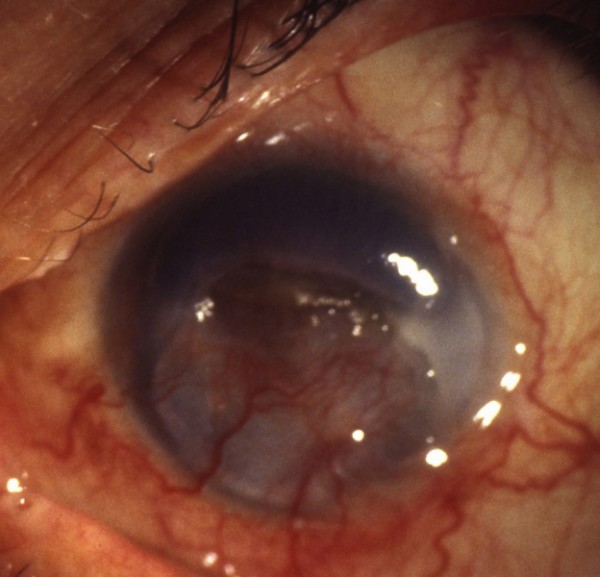
**Perforated Mooren's ulcer**.

A 74-year-old male presented at our clinic with a 10-day history of mild pain and loss of vision in his right eye. In 2003, he had undergone an exenteratio bulbi with the insertion of a prosthesis in the left eye following the corneal perforation of a Mooren's ulcer. Medical examination revealed acute conjunctival hyperaemia and a large perforated Mooren's ulcer in the right eye. The anterior chamber was absent and a seclusio pupillae with cataract were also present (Figure 1). Visual acuity was hand movements. Under general anaesthesia, we performed a 14 mm corneoscleral transplantation in association with the extracapsular extraction of the cataract, intraocular lens implantation and superior basal iridectomy.

The surgical technique included a total limbal peritomy in attempt to save limbal stem cells. A 14 mm trephine was used to mark and partially cut the scleral surface. After careful haemostasis, the anterior chamber was entered obliquely with a diamond knife and the sclera was cut with Castroviejo's corneal scissors. The entire cornea and scleral ring was removed, viscoelastic material was placed on the recipient bed and a large anterior capsulorhexis was performed. After the expression of the lens nucleus and aspiration of residual masses, an intraocular lens was implanted into the capsular bag and a peripheral iridectomy was performed. The donor tissue was trephined from a whole eye used within 24 hours of the donor's death and stored in a moist chamber using a trephine of the same diameter as that used on the recipient. The donor corneoscleral graft was then sutured into place onto the scleral ledge using 8.0 interrupted silk sutures. The anterior chamber was filled with viscoelastic material and the conjunctiva was closed with Vicryl 8.0 interrupted sutures.

**Figure 2:**
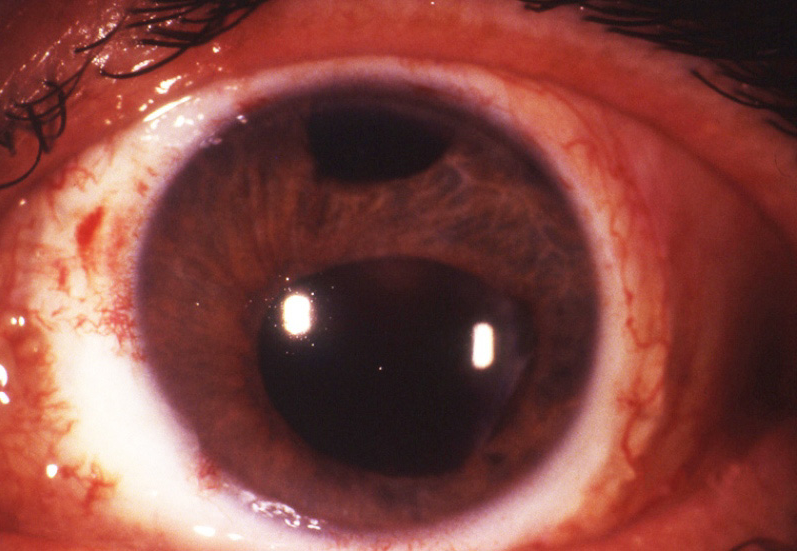
**Corneoscleral graft after six months**.

The day after surgery we found a mild keratitis with a low flare in the anterior chamber. The intraocular pressure measured with a pneumotonomer was 14 mm/Hg. Postoperative steroid therapy was prednisolone acetate 1% every two hours for four weeks (decreased to four times a day for five months), oral cyclosporine A (5 mg/kg/day) for six months and prednisone 25 mg a day for four months. After six months, the best corrected visual acuity was 6/60 (Figure 2).

## Conclusion

The presented case was a malignant form of bilateral Mooren's ulcer that had already caused a corneal perforation in the left eye with consequent phthisis bulbi. Local and systemic treatment with cortisone failed to prevent the progression of the disorder and perforation of the right eye and, therefore, a corneoscleral graft was necessary.

The etiopathogenesis of Mooren's ulcer is still unknown. It is probably an autoimmune disease. Indeed, anti-corneal and anti-conjunctival tissue antibodies have been isolated from patients suffering from this disorder [2]. Furthermore, the conjunctival tissue surrounding the lesion is generally rich in proteo-glycolytic enzymes secreted by mononucleate cells and neutrophils that progressively infiltrate the area surrounding the ulcer [3]. Another confirmation of the autoimmune origin of the disorder is the T-suppressor lymphocytes deficit in patient blood samples [4].

Wood and Kaufman classified Mooren's ulcer in two main forms [5]. Type 1 is the benign form, generally monolateral, which mainly affects the elderly. The symptoms are unclear but this type responds well to medical treatment and surgery. Type 2 is the malignant form. It can occur bilaterally in 25% of cases in white subjects and 75% in black subjects. It mainly affects the young. Watson divided the disease into three types based on the clinical presentation: unilateral Mooren's ulcer, bilateral aggressive Mooren's ulcer and bilateral indolent Mooren's ulcer [6].

In the third type of Watson and in the malignant form, we have a slowly progressive ulcer that affects not just the cornea but also the scleral tissue. For this reason, neither conventional medical nor surgical treatment is sufficient, and it can be necessary to perform a large corneal graft to preserve the anatomical integrity of the eye.

Indeed, it is extremely difficult to treat Mooren's ulcer and in many cases, the results are poor. Treatment starts with cortisone administered systemically and locally, but if this is unsuccessful, the complete excision of the perilimbal conjunctiva and episclera near the ulcer is made [6]. The use of immunosuppressive drugs, in particular cyclosporin A, should be reserved for more severe forms [7]. In the event of ulcer perforation, the area can be covered with amniotic membrane or a lamellar keratoplasty can be performed [8]. In this case, because of the presence of a large, perforated corneal ulcer affecting the scleral tissue, it was decided to perform a corneoscleral graft.

The use of this type of surgery to treat serious corneal disorders was first proposed in the early 1970s [9]. The percentage of surviving graft tissue has always been very low mainly because of the early onset of epithelial damage to the transplanted tissue, recurrence of the underlying disorder or secondary glaucoma caused by the disturbance of the iridocorneal angle and thereby of aqueous humour filtering [10].

In this case, the particular technique used made it possible to preserve the iridocorneal angle, and the administration of systemic and local cyclosporin treatment avoided the onset of secondary glaucoma as well as any sign of rejection six months after the operation. For this reason, we think that this type of graft can be proposed mainly for monocular patients with malignant Mooren's ulcer where there is serious damage to the corneal and scleral tissue and where eyes would otherwise be condemned to a complete loss of vision.

## Consent

Mauro Cellini, MD (Department of Surgery Science and Anesthesiology - Ophthalmology Service), who examined the patient, received informed written consent from the patient for the publication of the manuscript.

## Competing interests

The authors declare that they have no competing interests.

## Authors' contributions

MC performed the corneoscleral transplant and drafted the manuscript. MF recruited the patient from the Cornea Disease Service of the S.Orsola-Malpighi Hospital. ES reviewed the literature, CG examined the patient in the time and ECC reviewed the manuscript. All authors read and approved the final manuscript.
